# Diagnostic Imaging Methods and Comparative Analysis of Orbital Cavernous Hemangioma

**DOI:** 10.3389/fonc.2020.577452

**Published:** 2020-09-23

**Authors:** Li Zhang, Xuying Li, Fei Tang, Lu Gan, Xin Wei

**Affiliations:** West China Hospital, Sichuan University, Chengdu, China

**Keywords:** orbital cavernous hemangioma, radiography, computed tomography, magnetic resonance imaging, SPECT/CT, 99mTc-RBC

## Abstract

Orbital cavernous hemangioma is the most common primary tumor in the orbit. With the development of histopathology, it has been confirmed that cavernous hemangioma is not a real tumor, but a special type of vascular malformation. Cavernous hemangioma malformation(CVM) is a more appropriate way to name it. At present, surgical resection is the main treatment for CVMs. The prognosis of the surgery mainly depends on the location and the size of the lesion as well as its relationship with the optic nerve. Therefore, effective imaging information is of great importance. This paper analyzes the radiological imaging characteristics and the advantages and disadvantages between them, including ultrasound, CT, MRI, and SPECT/CT imaging of CVMs, hoping to help improve our understanding of CVMs.

## Introduction

Cavernous hemangioma malformation (CHM) is the most common primary benign orbital tumor in adults, which constitutes 6–9% of orbital lesions. In a retrospective study of 1,264 patients with suspected orbital tumor during 1971–2002, Shields et al. (1) found that CHMs constituted 6.12% of all lesions. Furthermore, Bonavolont et al. (2) analyzed 2,480 patients with orbital lesions; they found that CHM constituted 9% of all lesions. Finally, in a pathological analysis of 455 patients with orbital soft tissue tumors, Liu et al. (3) revealed that 258 patients had CHMs, which constituted 75.9% of orbital vascular tumors and 56.7% of orbital soft tissue tumors. The locations and sizes of these lesions vary among patients; they can compress adjacent structures and cause corresponding symptoms (e.g., vision loss, visual field defect, and eye movement disorder). Currently, there is a consensus regarding treatment of CHMs; specifically, when lesions are found in eyes with other normal characteristics, follow-up observation should be performed. The risk of surgery was found to be greater than any theoretical benefit for patients without visual impairment, as well as for patients in whom it was unclear whether the lesion was stable or actively growing. In contrast, surgery should be considered if the patient has a serious visual field defect at the time of initial visit, or if the symptoms are mild with signs of active growth (e.g., new symptoms, changes, or aggravation of original symptoms) (4). The main approaches for surgical treatment of CHMs include: transconjunctival approach, lateral approach, skin approach, and lateral combined medial conjunctiva. The methods, efficacy, and safety differ among these approaches (5, 6). Accurate preoperative qualitative and localization diagnosis are important prerequisites for successful surgery. With the improvement of people's consciousness of health and the physical examinations become universal, much more ocular diseases were detected in early stages. Accurately diagnosing CHM is of great significance for clinicians. Fully grasping the progression of the patients' conditions through images not only reduces the occurrence of serious complications such as vision loss, but help clinicians to deal with the lesions and surrounding tissues more confidently during the operation. Recently, studies regarding the histological and hemodynamic characteristics of CHMs have become increasingly in-depth, thus improving understanding regarding imaging manifestations of CHMs. Current diagnostic imaging methods for CHM mainly include ultrasound, computed tomography (CT), and magnetic resonance imaging (MRI). With the development of molecular imaging, radionuclide imaging technology has also been used for diagnosis of CHMs. The clinical characteristics, pathological characteristics, and imaging analysis are summarized in this review.

## Clinical Characteristics

CHMs are more common among women (60–70%), potentially because of hormone level (7). The onset age ranges from 17 to 86 years (mean, 45 years). Most CHMs are unilateral single lesions, while unilateral multiple lesions and bilateral lesions are extremely rare; however, the incidence of bilateral lesions may higher than expected (8). The most probable location is the lateral region of the posteromedial orbital space. The main clinical manifestation is painless chronic progressive exophthalmos. Cytokine and hormonal stimulation during puberty or pregnancy may cause acute exophthalmos or orbital enlargement. If the lesion oppresses the optic nerve or affects blood supply to the optic nerve, visual field defects may appear. In a few patients, monocular vision loss has been caused by orbital apex involvement. Other uncommon symptoms include pain, eyelid swelling diplopia, and palpable mass (1).

## Histopathological and Hemodynamic Characteristics

Histopathology has revealed that orbital cavernous hemangioma is not a real tumor. Its occurrence does not involve the proliferation of vascular endothelial cells, which suggests that it should be regarded as a unique type of vascular malformation. The components of the lesion mainly comprise veins, with a small number of small arterial vessels. In terms of blood flow mechanics, this lesion comprises a non-dilated arteriovenous malformation with low blood flow (9, 10). CHM is a tumor essence wrapped by a complete capsule, which is formed by outwardly extended fibrous tissue between vascular sinuses. Microscopy analysis demonstrates that the cavernous malformation is an expanded cavernous space, lined with flat endothelial cells. It is separated by fibrous intervals and surrounded by collagen trabeculae and smooth muscle cells. Occasionally, thrombus or hyaline degeneration are present, and calcification is rare (11).

## Radiological Imaging

### Conventional Ultrasound and Color Doppler Ultrasound (CDI)

Because of the complete capsule, the CHM boundary in ultrasound images has a clear margin and is slightly compressible. In addition, the CHM is composed of thin-walled enlarged blood sinuses with blood flow. Thus, we can see a large number of punctate echoes inside the tumors on B-scan. While on A-scan, it showed steep wave peak (indicating there is a capsule), regular medium high wave of internal reflection, 45°kappa angle (the imaginary angle between the internal reflection and the baseline reflection: the greater the angle, the more obvious the reduction), and moderate sound attenuation. Occasionally when it's difficult to distinguish CHM from schwannoma, the low reflection image of schwannoma on A-scan may help. Furthermore, blood in the blood sinuses is relatively static. Through CDI, no color blood flow signals or only a small number of punctate red and blue blood flow signals can be visualized (12–15). A study by Zhao et al. (16) revealed that slow compression of the eyeball during CDI causes most CHMs to exhibit rich red and blue blood flow signals; when partial pressure is maintained and no additional force is applied, the blood flow signals disappear. During slow relief of the pressure, the blood flow signals reappear; however, the color is distinct from that of pressurization (i.e., the original red signals become blue, while blue signals become red). This phenomenon is more obvious when the internal jugular vein is compressed. When the pressure is completely relieved, the blood flow signal disappears again. This process suggests that when the lesion is compressed, blood flow in the blood sinus is accelerated; this leads to the appearance of blood flow signals. In addition, when the pressure is maintained, either the blood sinus is blocked or blood flow recovers slowly, resulting in the disappearance of blood flow signals; when the pressure is relieved, blood rapidly fills the sinus cavity, which leads to reappearance of blood flow signals. The opposite blood flow signals during pressurization, compared with relief, indicate that revascularization of vascular sinuses is caused by venous return. This discovery provides novel clues for differential diagnosis of CHMs.

The advantages of ultrasound are its speed, non-invasive nature, and low cost; it is a very effective diagnostic method for CHMs. Nagaraju et al. (17) reported that the sensitivity, specificity, positive predictive value, negative predictive value, and accuracy of ultrasound in the diagnosis of ocular diseases were 94.2, 98.8, 99.1, 92.2, and 94.9%, respectively. However, there are notable limitations of this method. Ultrasound has limited value in detecting the size, location, and relationship of lesions, relative to surrounding structures. CDI imaging with compression is not obvious when lesions are deep or exhibit a complex location. Diagnosis may be difficult when lesions exhibit a complex internal composition, such as when they are accompanied by other vascular malformation components or inflammatory changes (e.g., when inflammatory cell infiltration occurs within the lesions). Furthermore, ultrasound is less useful for identification of lesions without extensive vasculature, such as schwannomas and pleomorphic adenomas (18).

### Ultrasonic Contrast

Also known as acoustic contrast, ultrasonic contrast is a technique that can enhance backscatter echo and improve the resolution, sensitivity, and specificity of ultrasound diagnosis using contrast agent. With the improvement of instrument performance and emergence of new acoustic contrast agents, ultrasonic contrast has increasing application in the diagnosis of ocular diseases, especially in retinochoroidal diseases. Because of the histological and hemodynamic characteristics of CHM, some clinicians have attempted to use contrast-enhanced ultrasound for its diagnosis. Ren et al. (19) reported that 49 of 52 patients (94.23%) diagnosed with CHMs showed typical enhancement patterns. Initial enhancement commonly appears at the periphery of the lesion, typically as small nodular protrusions. Occasionally, multiple nodular protrusions are fused; they slowly and gradually fill the lesion from the periphery to the center until reaching the peak of enhanced intensity, resulting in complete or incomplete filling of the whole lesion. After peak intensity is reached, it will be maintained for a short platform period, then gradually subside. This “snowball-like” dynamic enhancement pattern is the most characteristic manifestation of CHMs. Additionally, in ultrasonic contrast time-intensity curve, the enhancement pattern is slow onset and slow retreat (20). Contrast-enhanced ultrasound can directly reflect hemodynamic changes in the lesions; its advantages include absence of radiation, good repeatability, good patient tolerance, few adverse reactions, and usefulness for dynamic observation. However, thus far, ultrasonic contrast is more widely used in patients with chorioretinopathy, and there have been few studies regarding CHMs. Compared with traditional CT and MRI imaging, its advantages are unclear, and there remain inherent limitations of ultrasound imaging.

### CT

CT imaging has high-density resolution, which allows it to clearly show the size, position, and shape of the lesion, as well as its relationship with surrounding structures. Under conventional CT, most CHMs exhibit a similar appearance: predominantly located in the retrobulbar cone; oval or round shape; lobulated when enlarged; well-circumscribed; and homogeneous with smooth margins. In most patients, the transparent triangle area can be observed in the orbital apex. These features are not characteristic and have limited value in distinguishing CHM from neurofibroma, solitary fibroma, schwannoma, histiocytoma, and other tumors that may appear morphologically identical to CHMs. With the development of image technology, enhanced CT has enabled more detailed evaluation and identification of orbital lesions through different enhancement patterns, as well as better understanding of images with blood flow characteristics of the lesions. In contrast-enhanced CT images, CHMs exhibit poor and heterogeneous enhancement in early arterial and venous phases, due to their low arterial blood flow. Then, the contrast agent slowly accumulates and enhancement gradually increases, eventually resulting in persistent homogeneous enhancement in the late or delayed phase (21). This feature was confirmed in previous studies; Young et al. (22) also reported that most enhanced CT images of CHMs showed heterogeneous enhancement or incomplete filling in the late or delayed phase (88.5%). This phenomenon may result from large blood sinuses in the lesion while the blood supply arteries are relatively small, which causes slow circulation in the lesion. Drainage veins are larger than the nutrient arteries; thus, when contrast agent from blood supply arteries initially enters the lesion, a portion of the contrast agent is already discharged. Therefore, it exhibits heterogeneous progressive enhancement. Dynamic contrast-enhanced CT is a very accurate and useful method for diagnosis of CHMs; generally, it is sufficiently effective to enable differential diagnosis relative to other lesions (e.g., schwannoma). Young et al. (22) have divided CHMs into three types (i.e., low speed, medium speed, and fast speed) according to the contrast agent filling speed; they found that the unique early spot or patchy enhancement on contrast-enhanced CT was more favorable for distinguishing lesions such as schwannoma, compared with contrast-enhanced MRI, which may demonstrate a confusing early diffuse enhancement pattern. However, contrast-enhanced CT increases the radiation dose to patients, which has partially limited its applications. Clinicians should consider the corresponding risks during selection of imaging methods.

### MRI

As a popular imaging diagnosis method, MRI can detect and diagnose lesions by comparing signal strengths of differently weighted images, and obtain multi-directional images to clearly show tissues and structures as well as the anatomical relationships among them. Compared with CT, MRI can better display the relationships of orbital lesions with the optic nerve. Non-contrast MRI images of CHM are mainly manifested as follows: the lesion on T1-weighted images exhibits a signal hypointense to fat and isointense to extraocular muscle, while on T2-weighted images it exhibits a signal hyperintense to fat. This manifestation is difficult to distinguish CHMs from schwannoma and other lesions; thus, enhanced MRI is needed. On enhanced MRI, the enhancement pattern of lesions is similar to that of dynamic enhanced CT, with nodular enhancement in the early phase and continuous and homogeneous enhancement in the late and delayed phases. This feature can be used to distinguish isolated neurofibromas and schwannomas, which are characterized by diffuse heterogeneous or homogeneous enhancement in the early phase (21). However, Young et al. (22) found that in the early stage of contrast-enhanced MRI, one-fifth of CHMs exhibited diffuse and homogeneous enhancement, which was identical to that of schwannomas and other lesions. This differed from the results reported by Xian et al. (23), who found that early phase enhancement of CHMs always began from a small spot or a specific area. Young et al. (22) also found that in 43.3% of lesions, the enhancement was heterogeneous in the late or delayed phase, similar to the findings on dynamic enhanced CT. This phenomenon may be related to the slow internal blood flow of CHMs, which causes contrast agent to be unable to completely fill the lesions (even in the late or delayed phase). Nevertheless, MRI is of great value in the diagnosis of CHMs. In particular, using conventional MRI combined with enhanced MRI, the sensitivity was 79.4%; specificity was 100%; positive predictive value was 100%; negative predictive value was 83.7%; and accuracy was 90% (24). In quantitative dynamic magnetic resonance imaging, relevant parameters obtained based on the two-compartment dynamics model were further analyzed. With respect to the low internal blood flow characteristics of CHM and its abundant interstitium, as well as its relatively large extracellular space, the Ktrans (rate of contrast agent distribution from plasma to extracellular space of blood vessels) and Kep (rate at which contrast agents return from extracellular space to plasma) values were statistically significantly lower than those of schwannomas. Concurrently, resembling its enhancement pattern, the dynamic enhancement distribution pattern of the time intensity curve is mainly type I (i.e., continuously rising, such that signal strength increases slowly during observation; the curve exhibits a small ascending slope) (24–27). This feature aids in detection of the nature of the lesions and improves the reliability of diagnosis. However, the application of MRI also has limitations; in particular, patients with ferromagnetic implants or cardiac pacemakers, as well as patients with early pregnancy or claustrophobia, cannot undergo MRI. In addition, the gadolinium contrast agent used for contrast-enhanced MRI may cause renal systemic fibrosis in patients with renal insufficiency. Notably, accumulation of gadolinium contrast agent has been found during autopsies of individuals without renal dysfunction (28). In recent years, there has been increasing research regarding diffusion-weighted MRI, which is an important method for diagnosis of ischemic, infectious, and malignant diseases of the central nervous system. This non-invasive imaging method has a unique sensitivity for the free movement (diffusion) of water molecules in tissues. The apparent diffusion coefficient value of CHM is reportedly similar to that of solid tumors with medium density, which aids in differentiation from cystic lesions (28, 29). However, there have been few studies with relevant data and analysis regarding specific apparent diffusion coefficient thresholds for CHM, or regarding further differential diagnosis findings.

### 99mTc-RBC SPECT

Single-photon emission computed tomography (SPECT) is a modality used to achieve functional imaging of metabolic levels under pathological conditions, based on differences in blood circulation and tissue structure of lesions. It enables detection of cellular, molecular, and biochemical characteristics of lesions. Burroni et al. (30) reported that the characteristics of CHMs on SPECT imaging include the absence of intake during the early blood pool phase, followed by focal intake with persistent high consistency in the late phase. Additionally, false positive results were found within one hemangiopericytoma and one lymphangioma (both vascular diseases), which is consistent with the findings by Polite et al. (31); thus, more accurate CHM diagnosis is needed via combination with clinical manifestations, as well as CT and MRI manifestations. Nevertheless, the high sensitivity and specificity of SPECT imaging cannot be ignored. Dong et al. (32) performed further research regarding CHMs, using SPECT combined with CT imaging. They reported two false positive results regarding a venous hemangioma and a leiomyosarcoma with contrast agent concentration in late delayed imaging; the venous hemangioma was confirmed by hemorrhage and the leiomyosarcoma was confirmed by local bone destruction through postoperative pathological examination. Surprisingly, the sensitivity, specificity, accuracy, positive predictive value, and negative predictive value of radionuclide 99ctc-rbc SPECT plane imaging all reached 100% by further after-calibration analysis combined with visual analysis, semi-quantitative analysis, and receiver operating characteristic curve analysis (24). Although more large sample data are not available thus far, 99mTc-RBC SPECT plane imaging is considered to have very high diagnostic efficiency for CHMs. By combination with CT to provide anatomical information, the imaging accuracy of SPECT has been further improved; it even has the potential for use as a preoperative diagnostic modality for CHMs. However, limitations remain, such as poor diagnostic efficiency for other orbital lesions, relatively time-consuming nature, high cost, considerable equipment requirements, and radiation effects.

## Conclusion

Applications of ultrasound, CT, MRI, and 99mTc-RBC SPECT imaging in diagnosis for CHMs were discussed in this review. Ultrasonography is a non-invasive, rapid, and inexpensive diagnostic method; by combination with color Doppler ultrasound (based on internal lesion blood flow dynamics), ultrasonography can provide additional information regarding lesion characteristics, especially when using the method of eyeball compression. Although the diagnostic effectiveness is sufficient for CHMs, it may be difficult to make a fine diagnosis when the internal lesion structure is complex. In addition, the limited ability to supply information regarding anatomical structures is an inherent disadvantage of ultrasonography. To the best of our knowledge, there have been few studies regarding the application of contrast-enhanced ultrasound in CHM diagnosis, and we mainly cited from Chinese domestic literature. The characteristic “snowball-like” enhancement pattern for CHMs is an intuitive reflection of the internal hemodynamic properties of CHMs. CT and MRI are the most commonly used clinical imaging diagnostic techniques for orbital lesions. CT has better density resolution, while MRI has higher tissue resolution. However, both techniques have specific limitations in the differential diagnosis of CHMs. The imaging characteristics of lesions, such as schwannomas and pleomorphic adenomas, overlap with those of CHMs. Additional information regarding the internal nature of the lesion can be obtained through contrast-enhanced imaging. Furthermore, a more detailed comparison of enhancement patterns in the early phase and the late and delayed phases can facilitate accurate diagnosis. Additionally, the parameters of MRI dynamic enhancement and diffusion-weighted imaging provide further evidence for differential diagnosis. However, radiation, contrast agent allergy, and adverse reactions should be considered in the application of CT and contrast methods. Notably, 99mTc-RBC SPECT is considered molecular imaging; planar imaging reflects metabolism within the lesions. The different patterns and dosage of contrast agent intake are the basis of diagnosis. Based on combination with CT imaging, which provides anatomical information, the high diagnostic efficiency of 99mTc-RBC SPECT has made it a reliable choice for diagnosis of CHMs. However, when it is applied for other orbital lesions, instead of CHMs alone, the diagnostic efficiency decreases markedly; thus, it is a good choice for analysis of patients with high suspicion of CHMs, or when other imaging modalities are unavailable. With the rapid development of imaging diagnosis methods, the concept of diagnosing only based on experience is no longer applicable. Through the storage and analysis of a large number of different medical records, we can speak with data, and conduct quantitative and semi quantitative analysis based on relevant parameters of image data, including blood flow rate and enhancement degree, so as to make best diagnosis. With further developments in imaging technology, more accurate data-based criteria may become a trend in preoperative diagnosis.

## Author Contributions

XW brought up the idea and revised the manuscript. LZ collected information and wrote the manuscript. XL classified and sorted references for review. FT and LG helped with manuscript preparation and final version approval. All authors contributed to the article and approved the submitted version.

## Conflict of Interest

The authors declare that the research was conducted in the absence of any commercial or financial relationships that could be construed as a potential conflict of interest.
